# *Trypanosoma brucei* Secreted Aromatic Ketoacids Activate the Nrf2/HO-1 Pathway and Suppress Pro-inflammatory Responses in Primary Murine Glia and Macrophages

**DOI:** 10.3389/fimmu.2019.02137

**Published:** 2019-09-11

**Authors:** Nicole K. Campbell, David G. Williams, Hannah K. Fitzgerald, Paul J. Barry, Clare C. Cunningham, Derek P. Nolan, Aisling Dunne

**Affiliations:** ^1^School of Biochemistry and Immunology, Trinity College Dublin, University of Dublin, Dublin, Ireland; ^2^School of Medicine, Trinity Biomedical Biosciences Institute, Trinity College Dublin, Dublin, Ireland

**Keywords:** trypanosomes, keto acids, immune suppression, macrophages, glia

## Abstract

African trypanosomes, such as *Trypanosoma brucei* (*T. brucei*), are protozoan parasites of the mammalian vasculature and central nervous system that are best known for causing fatal human sleeping sickness. As exclusively extracellular parasites, trypanosomes are subject to constant challenge from host immune defenses but they have developed very effective strategies to evade and modulate these responses to maintain an infection while simultaneously prolonging host survival. Here we investigate host parasite interactions, especially within the CNS context, which are not well-understood. We demonstrate that *T. brucei* strongly upregulates the stress response protein, Heme Oxygenase 1 (HO-1), in primary murine glia and macrophages *in vitro*. Furthermore, using a novel AHADH^in^ *T. brucei* cell line, we demonstrate that specific aromatic ketoacids secreted by bloodstream forms of *T. brucei* are potent drivers of HO-1 expression and are capable of inhibiting pro-IL1β induction in both glia and macrophages. Additionally, we found that these ketoacids significantly reduced IL-6 and TNFα production by glia, but not macrophages. Finally, we present data to support Nrf2 activation as the mechanism of action by which these ketoacids upregulate HO-1 expression and mediate their anti-inflammatory activity. This study therefore reports a novel immune evasion mechanism, whereby *T. brucei* secretes amino-acid derived metabolites for the purpose of suppressing both the host CNS and peripheral immune response, potentially via induction of the Nrf2/HO-1 pathway.

## Introduction

Human African Trypanosomiasis (HAT), also known as African sleeping sickness, is caused by infection with the parasite *Trypanosoma brucei (T. brucei)*. There are two species of *T. brucei* which cause HAT; *Trypanosoma brucei gambiense*, which is responsible for 98% of HAT cases and results in a chronic disease course, and *Trypanosoma brucei rhodesiense*, which causes a rare zoonotic form of HAT with an acute disease course. *T. brucei* infection is transmitted by the tsetse fly, which alongside human and animal reservoirs complete the parasite's life cycle (1). Although the disease course of HAT can vary with *T. brucei* species, the disease is fatal in all cases unless treated. Despite recent encouraging developments, existing therapies for HAT remain strain and stage dependent. There are particular issues during the meningo-encephalitic stage with many drugs causing undesirable and often dangerous side effects or exhibiting a low therapeutic index. In addition, the emergence of drug resistance strains, and difficulties in administering intensive drug regimens in the rural and impoverished communities where the majority of HAT cases are located, all contribute to the need to develop new treatment strategies against *T. brucei* infection (2).

Although the immune system has multiple lines of defense against parasitic infections, *T. brucei* has developed mechanisms to avoid immune clearance, allowing it to persist as an exclusively extracellular parasite in the host and facilitate further transmission via the tsetse fly vector (3). The best studied immune evasion strategy employed by *T. brucei* is antigenic variation of the single variable surface glycoprotein (VSG) that covers the surface of the parasite (4). Macrophages act as one of the first lines of defense against *T. brucei* infection, with M1-type immune responses such as the production of pro-inflammatory mediators TNF-α and nitric oxide (NO) recognized as particularly important in parasitemia control [reviewed in (5)]. However, as strong immune responses pose a threat to the survival of trypanosomes and are potentially deleterious to the host, *T. brucei* acts to dampen the immune response in order to evade clearance by the immune system and promote host survival (3, 6). The second, meningo-encephalitic, stage of HAT occurs when *T. brucei* penetrates the blood brain barrier and is characterized by disturbances of the central nervous system (CNS) (2). It is unclear exactly why or how trypanosomes enter the brain, however it is known that immune activation of glial cells in the CNS occurs in response to trypanosome invasion (7–9). Despite the central contribution of the CNS invasion by *T. brucei* to the pathology and mortality of HAT, relatively little is known about how trypanosomes suppress the CNS immune response to facilitate their persistence in the brain and continued survival of the host (10).

Heme-oxygenase 1 (HO-1) is a stress-inducible enzyme which catalyzes the conversion of free heme to biliverdin and iron, with the concomitant release of carbon monoxide. Biliverdin can be further metabolized to bilirubin by biliverdin reductase. HO-1 and its products, biliverdin, bilirubin and CO, are well-known for their anti-inflammatory and anti-oxidant properties (11–15). Upregulation of HO-1 has been observed in certain parasitic infections, including *Plasmodium, Fasciola hepatica*, and *Leishmania chagasi* (16–18). Furthermore, expression of HO-1 has been associated with inhibition of the host immune response and parasite persistence (16–19). Interestingly, increased expression of HO-1 has also been observed in a model of *T. brucei* infection, however this has been attributed as a response to trypanosomiasis-associated anemia (3). How parasites such as *T. brucei* upregulate host HO-1 expression, and its consequences for the host immune response and survival, remains poorly understood.

It has long been recognized that trypanosomiasis is accompanied by a decrease in host circulating aromatic amino acids (tryptophan, tyrosine and phenylalanine) (20–25). This decrease occurs as a result of the constitutive uptake and subsequent transamination of aromatic amino acids by an unusual cytoplasmic aspartate aminotransferase (TbcASAT) in *T. brucei* (Supplementary Figure 1). This transamination reaction appears essential and results in the continuous production and excretion of aromatic ketoacids which can approach millimolar levels in circulation in infected animals (26–29). Interestingly, one of these aromatic ketoacids, indole pyruvate, derived from transamination of tryptophan, strongly suppressed LPS-induced pro-inflammatory cytokine IL-1β by macrophages (30). This result raised the possibility that trypanosomes secrete aromatic ketoacids within their hosts to lessen systemic pathologies associated with a persistent infection. However, anti-inflammatory effects for the other aromatic ketoacids, hydroxy-phenylpyruvate, and phenylpyruvate, derived from transamination of phenylalanine and tyrosine, respectively, have not been reported.

In this study, we explored this idea further and investigated the effects of aromatic ketoacids in both CNS and peripheral immune cells. We demonstrate that the trypanosome secretome strongly induces expression of the anti-inflammatory enzyme, HO-1, in glial cells. Using a novel *T. brucei* cell line which can metabolize aromatic ketoacids further to aromatic hydroxyacids, we have confirmed that trypanosome-generated aromatic ketoacids are not required for *T. brucei* growth, and that they mediate the induction of HO-1 in glia by the trypanosome secretome. Therefore, we report for the first time that the induction of HO-1 by *T. brucei* is mediated by trypanosome-derived aromatic ketoacids, and that this is achieved via Nrf2 activation. Furthermore, we demonstrate that these aromatic ketoacids are capable of inhibiting pro-inflammatory immune responses by glia and macrophages. Our study suggests a novel host-pathogen interaction whereby *T. brucei* secrete metabolites for the purposes of host immune suppression, via induction of the anti-inflammatory Nrf2/HO-1 pathway.

## Materials and Methods

### Reagents

The aromatic ketoacids indole pyruvic acid, hydroxy-phenylpyruvate and phenylpyruvate were purchased from Sigma Aldrich and dissolved in DMEM to a final concentration of 2 mM before use. Ultrapure lipopolysaccharide (LPS) from *E. coli* serotype O111:B4 was purchased from Invivogen.

### Mice

C57BL/6 mice were bred and housed under specific pathogen free conditions in the department of Comparative Medicine, Trinity College Dublin. All procedures were performed according to regulations and guidelines of the Trinity College Dublin Ethics Committee and under licensing of the Health Product Regulatory Authority (HPRA), Ireland.

### Glia Cultures

Whole brains were obtained from <1 day old C57BL/6 mice and dissected, chopped and placed in DMEM supplemented with 10% FCS, 2 mM L-glutamine, 100 U/ml penicillin, and 100 μg/ml streptomycin (all Sigma Aldrich). Tissue was triturated, the suspension was filtered through a sterile mesh (40 μm) and centrifuged (800 g, 5 min, 20°C). The cell pellet was resuspended and cultured in complete DMEM at 37°C in a 5% CO_2_ humidified environment for 12–14 days. After 24 h, media was replaced with complete DMEM containing granulocyte macrophage-colony stimulating factor (GM-CSF; 10 ng/ml, R&D Systems) and macrophage-colony stimulating factor (M-CSF; 20 ng/ml, R&D Systems), and replaced again every 3–4 days.

Non-adherent microglia were isolated by shaking (100 rpm, 2 h at room temperature), tapping and centrifuging (800 g, 5 min, 20°C). Remaining mixed glial cells were then removed by trypsin-EDTA digestion for 5 min, counted and plated at a concentration of 2.5 × 10^5^ cells/ml in complete DMEM.

### Bone Marrow Derived Macrophage Cultures

Primary bone marrow derived macrophages (BMDM) were obtained from the hind legs of adult C56BL/6 mice. Bone marrow from the tibiae and femurs was flushed out with complete DMEM and triturated. The cell suspension was centrifuged (300 g, 5 min, 20°C) and the cell pellet resuspended in ammonium chloride solution for 2 min to lyse red blood cells. Cells were centrifuged and resuspended in complete DMEM supplemented with 20% L929 medium containing M-CSF. Cells were seeded into petri dishes at a concentration of 5 × 10^5^ cells/ml, 10 ml per dish, and incubated at 37°C in a 5% CO_2_ humidified environment. After 3 days 1 ml of L929 medium containing M-CSF was added to each petri dish. Cells were harvested on day 6 by scraping adherent cells, which were then centrifuged (300 g, 5 min, 20°C), resuspended in complete DMEM supplemented with 10% L929 medium containing M-CSF and plated at 1 × 10^6^ cells/ml.

### Culture and Growth of *Trypanosoma brucei*

The species of *T. brucei* used in this study was the monomorphic, trypomastigote stage of *Trypanosoma brucei brucei*. The strain used was MITat 1.2, also termed Lister 427-2, that were modified to express T7 polymerase and Tet repressor elements and was maintained by selection in G418 (2.5 μg/ml) and hygromycin (5 μg/ml). Trypanosomes were cultured in sterile Hirumis' Modified Iscoves' Medium, formulation 9 (HMI-9), supplemented with 10% FCS, 180 mM NaHCO_3_, 1 mM β-mercaptoethanol, 50 mg/l ampicillin and streptomycin, at pH 7.5. Trypanosomes were cultured at 37°C in a 5% CO_2_ humidified environment. Cell growth density was maintained at a range between 1 × 10^5^ cells/ml and 2 × 10^6^ cells/ml.

### Generation of AHADH^in^ *T. brucei*

The related trypanosomatid *T. cruzi*, which causes American trypanosomiasis, does not secrete ketoacids which are instead metabolized further to the corresponding aromatic hydroxy acids by the enzyme L-alpha-hydroxyacid dehydrogenase (AHADH) (28, 29). In order to confirm that the ketoacids secreted by *T. brucei* mediate the observed increase in HO-1 expression in mixed glia treated with *T. brucei* supernatant, a novel *T. brucei* cell line with inducible AHADH expression was created. This conditional cell line was generated using p3859, a plasmid that allows tetracycline-inducible expression of transgenes (29). The complete open reading frame of AHADH (EMBL accession number AF112259, TriTrypDB gene ID TcCLB.506937.10) was amplified from *Trypanosoma cruzi* (*T. cruzi*) genomic DNA with the primers shown in Table 1 and subcloned into the pGEM-T vector. Positive clones were selected and sequenced. In order to remove the internal Not I restriction site present in the AHADH sequence, site-directed mutagenesis was performed on the cloned AHADH (in pGEM-T) using PCR and specific primers designed to mutate a single base of the AHADH NotI site (Table 1). Following DpnI digestion the amplification products were used to transfect *E. coli*, subsequently positive clones were selected and confirmed by digest/sequence analysis. The complete AHADH open reading frame was then excised from pGEM-T using a Hind III/BamH I double digest, and subcloned into Hind III/BamH I double digested p3859. Insertion of AHADH into the digested p3859 was confirmed by restriction digests and PCR. The plasmid was then subjected to Not I digestion, to allow targeting of the construct to the non-transcribed spacer in the rRNA locus, and used to transfect bloodstream forms *T. brucei*. Positive clones were selected for and maintained in complete HMI-9 supplemented with 5 μg/ml blasticidin, 2.5 μg/ml G418, and 5 μg/ml hygromycin.

**Table 1:** Primer sequences.

AHADH F	5′-GCGAAGCTTATGTTTTTTGAAGGTGCATGCGCGAAGGTG-3′
AHADH R	5′-CCGGATCCTTACAATGCCAAAGACAGCGACTCCGA-3′
AHADHNotIMuta F	5′-TCATTGCCGGAGGCCGCATGTTGG-3′
AHADHNotIMuta R	5′-CCAACATGCGGCCTCCGGCAATGAGGG-3′
NQO1 F	5′-GCTGCAGACCTGGTGATATT-3′
NQO1 R	5′-TGTAGGCAAATCCTGCTACG-3′
GSR F	5′-GGAAGCAGCCCTTCATCTTT-3′
GSR R	5′-TGGCAACTGTTCCTGAACTC-3′
β-Actin F	5′-GGACTCCTATGTGGGTGACGAGG-3′
β-Actin R	5′-GGGAGAGCATAGCCCTCGTAGAT-3′

### AHADH Assay

AHADH was used to detect the production of the ketoacids in a NADH coupled reaction as previously described (30). The decrease in absorbance at 340 nm was used to monitor the reaction; change in absorbance correlates directly to ketoacid production. The assay was performed using Tris buffer (25 mM, pH 7.4), NaCl (50 mM), NADH (0.25 mM), and AHADH (850 U), to a final volume of 1 ml.

### Viability Measurement

Viability of mixed glia and BMDM was measured by reduction of alamarBlue™ (BioRad) reagent. A volume of alamarBlue equal to 10% of the cell culture volume was added to each well and the plate was swirled gently to mix. Cells were incubated at 37°C for 6–18 h and then absorbance was read at 570 and 600 nm. The 600 nm absorbance values were subtracted from the 570 nm values, and cellular viability was expressed as a percentage of the untreated control.

### Western Blotting

For detection of HO-1 expression, mixed glia or BMDM were cultured in the presence of trypanosomes, trypanosome supernatants or ketoacids (0.25–1 mM) for 24 h. For detection of pro-IL-1β and iNOS mixed glia or BMDM were cultured in the presence of ketoacids (0.25–1 mM) for 30 min prior to stimulation with LPS (100 ng/ml) for 24 h. Cell lysates were prepared by washing cells in PBS prior to lysis in RIPA buffer (Tris 50 mM; NaCl 150 mM; SDS 0.1%; Na.Deoxycholate 0.5%; Triton X 100; all Sigma-Aldrich). For detection of Nrf2 mixed glia or BMDM were cultured in the presence of ketoacids (0.25–1 mM) for 6–24 h, then washed in PBS and lysed in Laemmli loading buffer. Samples were electrophoresed and transferred to PVDF membranes which were then blocked in 5% non-fat milk and incubated with monoclonal antibodies specific for HO-1 (Enzo Life Sciences), pro-IL-1β (R&D systems), iNOS or Nrf2 (both Cell Signaling) overnight at 4°C. Membranes were then washed in TBS-Tween and incubated with appropriate streptavidin-conjugated secondary antibody (anti-rabbit or anti-goat; both Sigma Aldrich) for 2 h at room temperature, prior to development with enhanced chemiluminescent substrate (Merck Millipore) using a BioRad ChemiDoc MP system. Subsequently, membranes were re-probed with HRP-conjugated monoclonal antibodies specific for β-actin (Sigma-Aldrich) as a loading control. Full length blots are presented in Supplementary Figures 4–8.

### Quantitative Real Time PCR

For detection of NQO1 and GSR expression by PCR mixed glia and BMDM were cultured in the presence of ketoacids (0.5–1 mM) for 24 h. RNA was extracted using the High Pure RNA Isolation Kit (Roche) and cDNA synthesized using High Capacity cDNA reverse transcription kit (Applied Biosystems). Quantitative real time PCR was carried out using iTaq Universal SYBR Green mastermix (BioRad) on a BioRad CFX96 Real-Time System. mRNA expression levels for NQO1 and GSR were quantified and were normalized to β-actin mRNA levels (primer sequences listed in Table 1).

### ELISA

For detection of cytokines, mixed glia, or BMDM were cultured in the presence of ketoacids (0.25–1 mM) for 30 min prior to stimulation with LPS (100 ng/ml) for 24 h. Concentrations of IL-6 and TNF-α were quantified from supernatants using R&D DuoSet ELISA kits (R&D Systems) or Ready-Set-Go ELISA kits (eBioscience) as per the manufacturers' protocols.

### Statistical Analysis

Statistical analysis was performed using Prism 6 software (GraphPad Software Inc.). Analysis of 3 or more data sets was performed by one-way ANOVA with Tukey's *post-hoc* test; *p*-values < 0.05 were considered significant and are denoted with asterisks in the figures.

## Results

### *Trypanosoma brucei* Secreted Factors Induce HO-1 Expression and Suppress Pro-Inflammatory Cytokines in Mixed Glia

**Figure 1:**
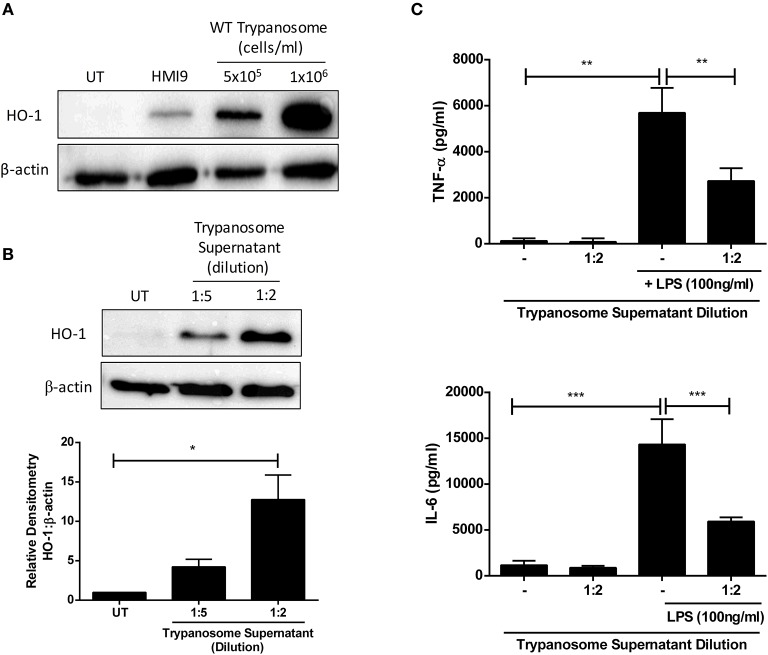
*Trypanosoma brucei* secreted factors induce HO-1 expression & suppress pro-inflammatory cytokines in mixed glia. **(A)** Primary murine mixed glia were cultured with different concentrations of *Trypanosoma brucei* for 24 h. Expression of HO-1 was measured by Western blot. Representative blot of three independent experiments is shown. **(B)** Supernatant from *Trypanosoma brucei* cultures was added to primary murine mixed glia. Expression of HO-1 was measured after 24 h by Western blot. Representative blot of three independent experiments is shown. Densitometric analysis of 3 immunoblots was performed using ImageLab (Bio-Rad) software. Results shown are mean ± SEM of the relative expression of HO-1: β-actin from 3 independent experiments. **(C)** Supernatant from *Trypanosoma brucei* cultures was added to primary murine mixed glia for 30 min prior to stimulation with LPS (100 ng/ml). Concentrations of TNFα and IL-6 in culture supernatants was measured by ELISA after 24 h. Results shown are mean ± SD concentrations from a triplicate culture, and are representative of three independent experiments. *p < 0.05, **p < 0.01, ***p < 0.001. Full length blots are presented in Supplementary Figure 4.

The anti-inflammatory and anti-oxidant stress-response enzyme HO-1 has been described to have immunosuppressive activity during parasitic infection (16–19). We first determined whether *T. brucei* can directly impact on HO-1 expression in primary murine mixed glia. *T. brucei* were co-cultured with mixed glia at different concentrations for 24 h, and HO-1 expression was assessed by Western blot. It was observed that mixed glia cultured with *T. brucei* strongly upregulated HO-1 expression compared to mixed glia cultured in media alone (Figure 1A). In order to determine whether upregulation of HO-1 is mediated by direct contact between mixed glia and trypanosomes, or by trypanosome-secreted factors, supernatants from cultures of *T. brucei* were added to mixed glia at a 2- and 5-fold dilution for 24 h and HO-1 expression was assessed by Western blot. A dose-dependent upregulation of HO-1 was observed in mixed glia treated with *T. brucei* supernatant (Figure 1B). HO-1 expression was also assessed using trypanosomes grown under serum free conditions in order to rule out the possibility that factors contained in FCS are driving HO-1 expression. We found that removal of 10% FCS supplement leads to lower secreted ketoacid levels (Supplementary Figure 2A). This is also reflected by slightly lower HO-1 levels, however there is still a clear increase in HO-1 expression in glia that were treated with the trypanosome supernatant vs. cells that were treated with media containing no trypanosome supernatant (Supplementary Figure 2B). Additionally, we assessed whether *T. brucei* supernatant could suppress pro-inflammatory cytokine production by mixed glia. Cells were treated with *T. brucei* supernatant for 30 min prior to stimulation with LPS for 24 h, and the concentration of IL-6 and TNFα was measured by ELISA. The *T. brucei* culture supernatant significantly reduced the production of both cytokines following LPS-stimulation (Figure 1C).

### *Trypanosoma brucei* Secreted Aromatic Ketoacids Are Non-toxic and Induce HO-1 Expression in Mixed Glia and Macrophages

**Figure 2:**
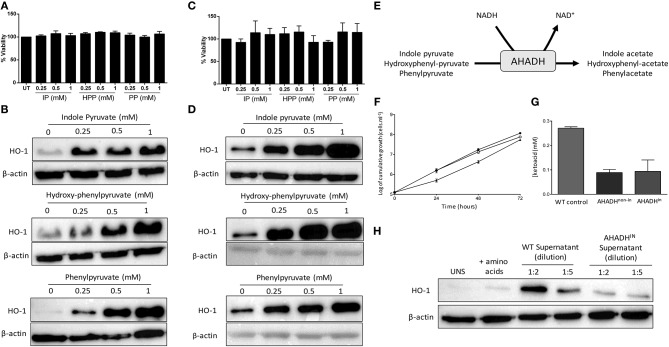
*Trypanosoma brucei* produce and secrete aromatic ketoacids which are non-toxic and induce HO-1 expression in mixed glia and BMDM. Primary murine mixed glia and BMDM were incubated with indole pyruvate, hydroxyl-phenyl pyruvate, or phenylpyruvate (0.25–1 mM) for 24 h. **(A)** Viability of mixed glia was determined by alamarBlue reduction, and is expressed as a percentage of the untreated control. Results shown are mean ± SEM of the percentage viabilities of mixed glia from three independent experiments. **(B)** Expression of HO-1 in mixed glia was determined by Western blot. Blots shown are representative of three independent experiments. **(C)** Viability of BMDM was determined by alamarBlue reduction, and is expressed as a percentage of the untreated control. Results shown are mean ± SEM of the percentage viabilities of BMDM from four independent experiments. **(D)** Expression of HO-1 in BMDM was measured by Western blot. Blots shown are representative of two independent experiments. **(E)** Schematic depicting metabolism of aromatic ketoacids to aromatic hydroxyacids by AHADH. **(F)** Bloodstream form AHADH^in^ cells were found to grow at similar rates to wild type MITat 1.2 *T. brucei* (▴), whether induced (°) or non-induced (•). **(G)** Secreted aromatic ketoacid concentrations in HMI-9 culture media, as measured via AHADH assay after 48 h. AHADH cells were either non-induced or induced with 2 mg/ml tetracycline. Results shown are mean ± SD of triplicate measurements, and are representative of three independent experiments. **(H)** Supernatants from WT and AHADH^in^ *T. brucei* were added to mixed glia for 24 h. HO-1 expression was measured by Western blot. Representative blot of three independent experiments is shown. Full length blots are presented in Supplementary Figure 5.

It has previously been reported that *T. brucei* secretes the aromatic ketoacids indole pyruvate, hydroxy-phenylpyruvate, and phenylpyruvate, and that indole pyruvate can inhibit pro-IL-1β expression in BMDM via HIF1α destabilization (25, 27, 30). Having confirmed that *T. brucei* induces HO-1 expression in mixed glia, and that induction is mediated by factors secreted by the trypanosome, we next determined whether these aromatic ketoacids can directly upregulate HO-1 expression in both mixed glia and BMDM. Viability assays were carried out to ensure that the aromatic ketoacids are non-toxic to mixed glia and BMDM at the concentrations used in our study, which mimic typical ketoacid concentrations observed during *in vivo* trypanosomiasis (30). Mixed glia and BMDM were cultured with indole pyruvate, hydroxy-phenylpyruvate, or phenylpyruvate (0.25–1 mM) for 24 h, after which cellular viability and HO-1 expression was assessed. All three ketoacids were found to be well-tolerated and non-toxic to mixed glia (Figure 2A) or BMDM (Figure 2C). Furthermore, all three ketoacids induced a strong upregulation of HO-1 expression in both cell types (Figures 2B,D). This effect was dose-dependent, with indole pyruvate and hydroxy-phenylpyruvate showing greater potency compared to phenylpyruvate. Removal of serum from the media did not affect the ability of indole pyruvate or hydroxyphenylpyruvate to induce HO-1 expression in BMDM. However, only a very weak induction of HO-1 was observed by phenylpyruvate when media was replaced with serum free media, suggesting that serum components may impact on the solubility of this ketoacid (Supplementary Figure 2C).

The related Trypanosomatid *T. cruzi* expresses an NAD-linked aromatic α-hydroxy acid dehydrogenase (AHADH) that catalyzes the reduction of aromatic ketoacids to the corresponding hydroxyacid (Figure 2E) (31). Consequently, a conditional AHADH *T. brucei* cell line was generated in order to determine whether secretion of ketoacids was essential to *T. brucei* growth and to produce a bloodstream form that would secrete less ketoacids. This conditional cell line was generated using p3859, a plasmid that allows tetracycline-inducible expression of transgenes (29). Both non-induced and AHADH-induced *T. brucei* had comparable growth rates (Figure 2F). Significantly, both non-induced and induced AHADH(in) cells were found to have similar decreased levels of aromatic ketoacids in their culture media, which could be due to leaky expression of the AHADH(in) vector in the absence of tetracycline. In both cases there was a ~60–70% reduction in ketoacid secretion compared to the wild type parental cell line with wild-type *T. brucei* producing ~2.5 times more keto acid than mutant strain (Figure 2G). These data show that production of aromatic ketoacids is not an essential process and that, at least in these cell lines, expression of the AHADH activity was effectively constitutive and not subject to tetracycline regulation. Having confirmed the AHADH^in^ *T. brucei* cell line exhibited reduced ketoacid secretion, and showed no growth deficiency, supernatants from wild type and AHADH^in^ *T. brucei* were added to mixed glia cultures. After 24 h, HO-1 expression was measured by Western blot. As previously observed, wild type *T. brucei* supernatant strongly upregulated HO-1 expression in mixed glia. However, the AHADH^in^ *T. brucei* supernatant displayed a dramatically reduced capacity to induce HO-1 expression in these cells. Clear differences were seen when both the wild-type and AHADH^in^ *T. brucei* supernatants were diluted 2-fold, while comparable levels of HO-1 were expressed in cells treated with the wild type *T. brucei* supernatant (diluted 1:5) and AHADH^in^ *T. brucei* supernatant (diluted 1:2) as the overall keto acid concentration in each case should be approximately equal (Figure 2H).

### Aromatic Ketoacids Exhibit Anti-inflammatory Activity in Primary Glia and Macrophages

**Figure 3:**
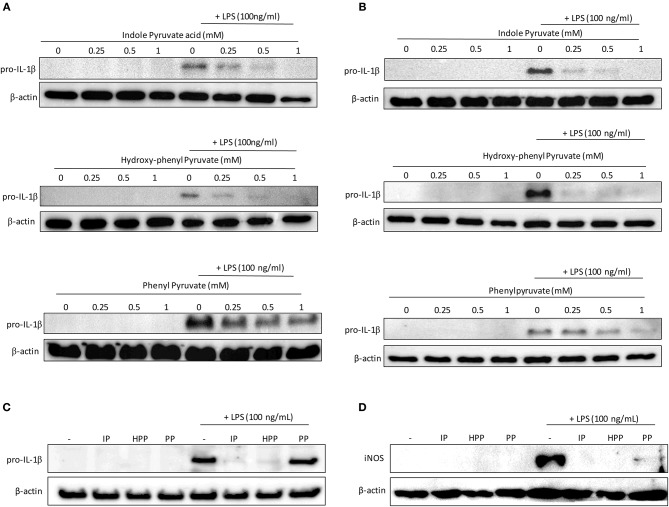
Ketoacids inhibit pro-IL-1β expression in LPS-stimulated mixed glia, microglia, and BMDM. Primary murine mixed glia, microglia and BMDM were treated with indole pyruvate (IP), hydroxy-phenylpyruvate (HPP), or phenylpyruvate (PP) (0.25–1 mM) for 30 min prior to stimulation with LPS (100 ng/ml) for 24 h. Expression of pro-IL-1β in **(A)** mixed glia, **(B)** microglia, and **(C)** BMDM was measured by Western blot. Blots shown are representative of two to three independent experiments. Expression of iNOS in **(D)** BMDM was measured by Western blot. Blots shown are representative of three independent experiments. Full length blots are presented in Supplementary Figure 6.

Having confirmed that the *T. brucei*-derived ketoacids, indole pyruvate, hydroxy-phenylpyruvate, and phenylpyruvate can upregulate HO-1 expression in mixed glia and BMDM, and given that HO-1 has well-established anti-inflammatory properties, we sought to determine if the ketoacids themselves can serve as anti-inflammatory molecules. It has previously been reported that indole pyruvate inhibits production of the highly pro-inflammatory cytokine, IL-1β, by macrophages (30), therefore we investigated whether the other ketoacids hydroxy-phenylpyruvate and phenylpyruvate had similar activity, and whether this effect would be observed in mixed glia as well as macrophages. Microglia were also included in this experiment given their macrophage-like role in the brain. Mixed glia, microglia and BMDM were treated with ketoacids for 30 min prior to stimulation with LPS. After 24 h the expression of the pro-form of IL-1β, pro-IL-1β, was detected by Western blot. Both indole pyruvate and hydroxy-phenylpyruvate strongly inhibited pro-IL-1β expression by mixed glia at all concentrations tested, while phenylpyruvate reduced pro-IL-1β only at higher concentrations (Figure 3A). Similarly, indole pyruvate and hydroxy-phenylpyruvate treatment abrogated pro-IL-1β expression in microglia and BMDM, while phenylpyruvate had no effect (Figures 3B,C).

Inducible nitric oxide synthase (iNOS) catalyzes the production of NO from L-arginine, and contributes to pro-inflammatory responses in glia and BMDM (31, 32). In order to examine whether ketoacids could modulate iNOS expression, BMDM, and mixed glia were pre-treated with ketoacids and stimulated with LPS as before. After 24 h iNOS expression was measured by western blot. Expression of iNOS was below the limit of detection in mixed glia however, the enzyme was upregulated in LPS-stimulated BMDM (Figure 3D). As was the case with pro-IL-1β, iNOS expression was abrogated in the cells upon treatment with the ketoacids.

**Figure 4:**
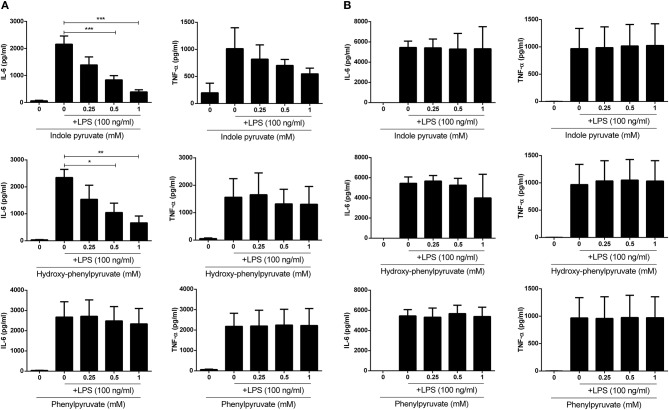
Indole pyruvate and hydroxyl-phenyl pyruvate inhibit pro-inflammatory cytokine production in LPS-stimulated microglia but not BMDM. Primary murine microglia and BMDM were treated with indole pyruvate, hydroxy-phenylpyruvate, or phenylpyruvate (0.25–1 mM) for 30 min prior to stimulation with LPS (100 ng/ml) for 24 h. Concentrations of TNFα and IL-6 in **(A)** mixed glia and **(B)** BMDM supernatants was measured by ELISA. Results shown are mean ± SEM concentrations of IL-6 and TNFα from three to six independent experiments. ***p < 0.001, **p < 0.01, *p < 0.05.

Having observed reduced expression of pro-IL-1β upon ketoacid treatment, we next examined whether they were also capable of reducing expression of the pro-inflammatory cytokines, IL-6 and TNFα, in primary glia and BMDM. Mixed glia and BMDM were pre-treated with ketoacids and stimulated with LPS as before and the concentration of IL-6 and TNFα in culture supernatants was measured by ELISA. Both indole pyruvate and hydroxy-phenylpyruvate significantly reduced IL-6 and TNFα production by mixed glia, and this inhibition was dose dependent. Phenylpyruvate did not reduce either IL-6 or TNFα production by mixed glia (Figure 4A). Conversely, none of the ketoacids tested had any effect on IL-6 or TNFα production by BMDM (Figure 4B). Attempts were made to make a direct link between HO-1 induction and cytokine inhibition by the ketoacids, however this proved technically difficult given the challenge of simultaneously inducing and inhibiting the HO-1 protein, as has been previously reported (33). High doses of HO-1 inhibitors and siRNA were employed in an attempt to counter the potent induction of HO-1 by the ketoacids, but this unfortunately reduced the viability of our cells, particularly when used in combination with indole pyruvate and phenylpyruvate treatment. We did however observe that inhibition of HO-1 partially restored LPS-induced pro-IL-1β expression in hydroxyphenylpyruvate treated BMDM (Supplementary Figure 3). Further work in HO-1 deficient cells is required to confirm the direct link between HO-1 induction and cytokine inhibition by the ketoacids.

### Ketoacids Activate Nrf2 in Mixed Glia and BMDM

**Figure 5:**
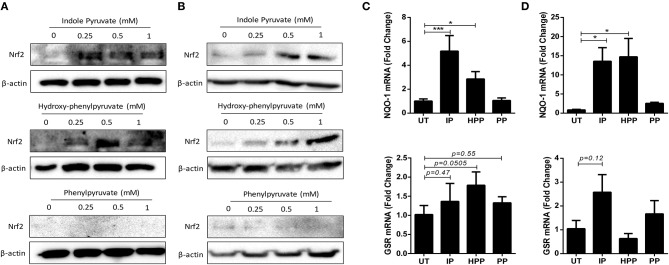
Ketoacids induce Nrf2 expression and upregulate Nrf-2 dependent genes in mixed glia and BMDM. **(A)** Primary murine mixed glia were treated with indole pyruvate, hydroxy-phenylpyruvate, or phenylpyruvate (0.25–1 mM) for 24 h. Expression of Nrf2 was measured by Western blot. Blots shown are representative of three independent experiments. **(B)** BMDM were treated with indole pyruvate, hydroxy-phenylpyruvate or phenylpyruvate (0.25–1 mM) for 24 h. Expression of Nrf2 was measured by Western blot. Blots shown are representative of three independent experiments. **(C)** Primary murine mixed glia were treated with indole pyruvate (IP), hydroxy-phenylpyruvate (HPP), or phenylpyruvate (PP) (0.5 mM) for 24 h. mRNA expression of NQO-1 and GSR was measured by RT-PCR. **(D)** BMDM were treated with indole pyruvate, hydroxy-phenylpyruvate, or phenylpyruvate (1 mM) for 24 h. mRNA expression of NQO-1 and GSR was measured by RT-PCR. Results shown are mean ± SEM fold expression of NQO-1 and GSR from three independent experiments. ***p < 0.001, *p < 0.05. Full length blots are presented in Supplementary Figure 7.

Finally, to elucidate the mechanism by which *T. brucei* derived ketoacids upregulate HO-1 in mixed glia and BMDM, we next investigated whether the ketoacids activate the transcription factor nuclear factor (erythroid-derived 2)-like 2 (Nrf2) which regulates the expression of a number of anti-oxidant genes, and is the primary regulator of HO-1. Under steady-state conditions Nrf2 is bound to Keap1 and targeted for degradation; however, during oxidative stress Nrf2 is released from Keap1 and can migrate to the nucleus (34). To measure Nrf2 accumulation, mixed glia and BMDM were treated with ketoacids for up to 24 h, and Nrf2 was detected by Western blot. Both indole pyruvate and hydroxy-phenylpyruvate dose-dependently increased Nrf2 accumulation in mixed glia (Figure 5A) and BMDM (Figure 5B). Nrf2 expression was not detected with phenylpyruvate in either cell type. To confirm activation of the Nrf2 pathway, we measured expression of additional Nrf2-regulated genes following ketoacid treatment. Mixed glia and BMDM were treated with ketoacids for 24 h, after which the expression of NAD(P)H dehydrogenase (quinone 1) (NQO-1) and glutathione reductase (GSR), was measured by RT-PCR. In mixed glia, indole pyruvate and hydroxy-phenylpyruvate both significantly increased expression of NQO-1, and hydroxy-phenylpyruvate also showed a trend toward increased GSR expression. Phenylpyruvate did not upregulate expression of either gene (Figure 5C). Similarly, in BMDM, both indole pyruvate and hydroxy-phenylpyruvate significantly upregulated NQO-1 expression, while only indole pyruvate showed a trend toward increased GSR expression, and phenylpyruvate again had no effect on either gene (Figure 5D).

## Discussion

HAT is a chronic parasitic disease, associated with considerable morbidity and mortality, that is caused by African trypanosomes. The challenge facing trypanosomes is to maintain a persistent infection while simultaneously prolonging host survival in order to potentiate parasite transmission to the tsetse fly vector to ensure completion of the life cycle. This challenge is of particular relevance for trypansomes which are exclusively extracellular and, unusually for a pathogen, can invade the CNS. Here we show that *T. brucei* can upregulate expression of the anti-inflammatory stress-response enzyme HO-1 within host CNS and peripheral immune cells. Significantly, all three aromatic ketoacids secreted by *T. brucei*, indole pyruvate, hydroxy-phenylpyruvate, and phenylpyruvate, can mediate this induction of HO-1. Therefore, our results not only describe a previously unknown function for the production of aromatic ketoacids by *T. brucei*, but also a novel host-pathogen interaction whereby *T. brucei* has evolved to secrete metabolites designed to promote an overall anti-inflammatory response in the host.

Although upregulation of HO-1 expression has been observed in other parasitic infections (16–18), relatively little is known about the role of HO-1 in trypanosomiasis. Expression of HO-1 has been observed in a murine model of *T. cruzi* infection by Gutierrez et al. who also examined the effect of HO-1 activity on the host immune response and survival. In their study, treatment with a HO-1 inhibitor exacerbated inflammation and limited host survival, while conversely a HO-1 inducer reduced production of pro-inflammatory cytokines and promoted anti-inflammatory responses, including production of IL-10 and induction of Tregs (19). In the present study, we observed a strong upregulation of HO-1 in glial cells cultured with *T. brucei*. In agreement with this observation, Stijlemans et al. have reported increased expression of HO-1 within a murine model of *T. brucei* infection (5). Additionally, they have reasoned this upregulation of HO-1 as a response to anemia resulting from the accumulation of iron by trypanosomes (35). As it is currently unknown whether *T. brucei* themselves express the cellular machinery necessary to extract iron from heme-containing proteins (36), it is tempting to speculate that they have evolved to upregulate HO-1 in their host for the purposes of both increasing their supply of iron and creating a trypanotolerant immune environment.

Despite reports that increased HO-1 expression is a feature of parasitic infections, it is unknown how parasites including trypanosomes achieve this upregulation of HO-1 in their host. To clarify how *T. brucei* mediates its upregulation of HO-1, we investigated whether a factor secreted by the trypanosome may be responsible for this effect. We found that treatment of mixed glia with *T. brucei* supernatant produced a similar upregulation of HO-1 expression, and additionally suppressed pro-inflammatory cytokine production. This observation is in agreement with a previous study by Garzón et al. who reported that treatment of murine BMDC with *T. b. gamiense* secretome effectively limited DC maturation and pro-inflammatory functions (37). However, the specific factors present in the *T. b. gamiense* secretome which mediate these anti-inflammatory effects have not been identified. It is known that *T. brucei* infections are associated with disturbed aromatic amino acid profiles, owing to the metabolism of aromatic amino acids into aromatic ketoacids by *T. brucei* (25–27). To date, however, there has been limited research identifying the purpose of these ketoacids. It has recently been reported that one of these ketoacids, indole pyruvate, has anti-inflammatory effects in BMDM (30). We therefore hypothesized that ketoacids could be factors secreted by *T. brucei* which mediate our observed anti-inflammatory effects, via induction of HO-1. To test this hypothesis we treated both BMDM and glia with three different ketoacids, indole pyruvate, hydroxyl-phenyl pyruvate, and phenylpyruvate, and found that all three were capable of strongly upregulating HO-1 expression. Furthermore, we created a novel *T. brucei* cell line which possesses inducible expression of the ADADH enzyme, which catalyzes the conversion of ketoacids to hydroxyacids. Using this ADADH^in^ *T. brucei* we were able to confirm that aromatic ketoacids are mediators of HO-1 upregulation within the *T. brucei* secretome. To our knowledge, the present study is the first to identify *T. brucei* derived ketoacids as HO-1 inducers. Further study using cASAT deficient trypansosomes will determine if other *T. brucei* secreted factors are also capable of inducing this stress response pathway. In addition, future studies will determine the precise concentration of the individual keto acids produced by the parasite, given that the ADADH enzyme assay which is used to measure overall ketoacid production does not distinguish between indole pyruvate, phenylpyruvate, and hydroxyphenyl- pyruvate.

While there have been previous studies investigating evasion by *T. brucei* of the peripheral immune system, studies examining the effects of *T. brucei* on the CNS immune response have been scarce, presumably due to the difficulties inherent in accessing CNS cells. Nonetheless, this presents a significant obstruction to our understanding of HAT, especially considering that invasion of the CNS by *T. brucei* is an important clinical event in the course of this disease. In order to further our knowledge of immune evasion by *T. brucei* in the CNS, we elected to use primary murine glial cells, as well as BMDM, in the present study. Following our earlier experiments demonstrating that *T. brucei* supernatant inhibits pro-inflammatory cytokine production by glial cells, and our identification of aromatic ketoacids as *T. brucei* derived factors responsible for the upregulation of HO-1, we performed further experiments to test whether these ketoacids also possess anti-inflammatory activity in glia and BMDM. We found that all three ketoacids produced anti-inflammatory effects, with indole pyruvate and hydroxy-phenylpyruvate displaying greater activity over phenylpyruvate. In both glia and BMDM we observed a marked reduction in pro-IL-1β, along with a reduction in iNOS expression in BMDM, in response to LPS stimulation with ketoacid treatment. Interestingly, while we observed a significant reduction in IL-6 and TNFα production by glia treated with indole pyruvate and hydroxy-phenylpyruvate, no reduction of these cytokines was seen in BMDM treated with either ketoacid. This result is in agreement with a previous report that indole pyruvate reduced IL-1β, but not IL-6 or TNFα, in murine macrophages (30). This divergence in responses between glia and BMDM is very interesting and may reflect the greater need to avoid immune activation or inflammation within the CNS during trypanosomiasis in order to prolong host survival and, therefore, persistence of the parasite. Indeed, in a model of *T. brucei* infection, treatment with antagonists of IL-1β and TNFα attenuated neurodegeneration, highlighting the significant role these pro-inflammatory cytokines play in trypanosomiasis-associated neuropathology (38). Furthermore, HO-1 upregulation has been shown to be protective during neurological insults. For example, induction of HO-1 has been reported to improve outcomes in models of autoimmune neuroinflammation (39) and stroke (40). Further research is required to identify the mechanism(s) underlying the difference in responses to *T. brucei* derived ketoacids between glia and macrophages. It will also be of interest to determine if levels of ketoacids in the CNS are comparable to those seen in the circulation during trypanosomiasis.

Finally, to further understand how the ketoacids upregulate HO-1 expression in glia and BMDM we investigated upstream engagement of the transcription factor Nrf2. Nrf2 is typically activated under conditions of oxidative stress, and promotes the expression of anti-oxidant proteins including HO-1, NQO-1, and GSR. We found that both indole pyruvate and hydroxy-phenylpyruvate treatment stabilized Nrf2 expression, and increased expression of NQO-1 and GSR mRNA, in both glia and BMDM. We were unable to detect increased expression of Nrf2, NQO-1, or GSR with phenylpyruvate treatment, which may reflect its less potent induction of HO-1 and anti-inflammatory activity compared to the other ketoacids. Activation of the Nrf2 pathway has been described as an important regulator of the stress response in parasitic infections, which protects the host from deleterious immune activation, at the expense of persistence of the infection (41). The role of Nrf2/HO-1 in *T.cruzi* infection has been highlighted by Paiva et al. who report that the suppression of oxidative stress by this pathway promotes parasitemia (42). Interestingly, Aoki *et al*. have reported that the aromatic ketoacids protect against UV-B induced skin damage, by reducing cytotoxicity and the production of IL-1β and IL-6 (43). Both Nrf2 and HO-1 have been reported as protective against the oxidative damage caused by UV radiation (44–46). Further work is required to identify what role the Nrf2/HO-1 pathway plays in *T. brucei* infection, and how ketoacids produced by *T. brucei* activate Nrf2.

In conclusion, we have presented convincing data to support a hitherto-unidentified role for the *T. brucei* derived aromatic ketoacids in host immune evasion, which involves activation of the stress response Nrf2/HO-1 pathway. This immunosuppressive mechanism appears to be effective vs. immune responses in both the CNS and periphery, however, the effects produced are cell-type dependent. It is striking that aromatic ketoacids secreted by bloodstream forms of *T. brucei* can promote an anti-inflammatory response while simultaneously suppressing production of pro-inflammatory cytokines by immune cells. Further research is required to confirm the overall anti-inflammatory effects of these ketoacids *in vivo* and their link with activation of Nrf2/HO-1. It is hoped that future research in this area can yield new tools to treat HAT, and perhaps utilize aromatic ketoacids as treatments for other inflammatory diseases.

## Author Summary

African trypanosomes, such as *Trypanosoma brucei* (*T. brucei*) are protozoan parasites best known for causing Human African Trypanosomiasis (HAT) which, if untreated, is fatal. Current therapies have limitations, while vaccination or prophylactic intervention is not possible. Trypanosomes are exclusively extracellular and evade the immune defenses by constantly changing the single protein that covers their surface. This mechanism results in characteristic waves of parasitemia as the parasite numbers rise and then fall following host antibody mediated lysis only for a new antigenic wave to emerge. The parasite load is huge and their clearance repeatedly releases large amounts of parasite material systemically with the potential to drive harmful host innate and inflammatory responses. Here we report a previously unknown mechanism by which *T. brucei* can suppress these responses. We show that aromatic ketoacids, derived from amino acids that are constitutively secreted by the parasite into the host, are potent inducers of the anti-inflammatory and stress response enzyme, Heme Oxygenase 1 (HO-1). We also show that these same ketoacids reduce inflammation in both peripheral and CNS immune cells. Our study provides new insight into how *T. brucei* manipulates the host immune system and persists in the host. Increased understanding of how ketoacids upregulate HO-1, and what their role is during HAT, may pave the way for the development of new treatments for HAT and other inflammatory diseases.

## Data Availability

All datasets generated for this study are included in the manuscript/Supplementary Files.

## Ethics Statement

The animal study was reviewed and approved by all procedures were performed according the regulation, guideline, and under licensing of the Health Product Regulatory Authority (HPRA), Ireland.

## Author Contributions

NC, DW, PB, DN, and AD conceptualized and designed experiments. NC, DW, HF, and CC performed experiments. PB and DN designed and created the AHADH^in^ *T. brucei* cell line. NC, HF, DN, and AD wrote the manuscript.

### Conflict of Interest Statement

The authors declare that the research was conducted in the absence of any commercial or financial relationships that could be construed as a potential conflict of interest.

## References

[1] Franco JR Simarro PP Diarra A Jannin JG. Epidemiology of human African trypanosomiasis. Clin Epidemiol. (2014) 6:257–75. 10.2147/CLEP.S39728 25125985 PMC4130665

[2] Stich A Abel PM Krishna S. Human African trypanosomiasis. Br Med J. (2002) 325:203–6. 10.1136/bmj.325.7357.203 12142311 PMC1123723

[3] Stijlemans B Caljon G Van Den Abbeele J Van Ginderachter JA Magez S De Trez C. Immune evasion strategies of *Trypanosoma brucei* within the mammalian host: Progression to pathogenicity. Front Immunol. (2016) 7:233. 10.3389/fimmu.2016.00233 27446070 PMC4919330

[4] Borst P. Antigenic variation and allelic exclusion. Cell. (2002) 109:5–8. 10.1016/S0092-8674(02)00711-0 11955440

[5] Stijlemans B Guilliams M Raes G Beschin A Magez S De Baetselier P. African trypanosomosis: from immune escape and immunopathology to immune intervention. Vet Parasitol. (2007) 148:3–13. 10.1016/j.vetpar.2007.05.005 17560035

[6] Paulnock DM Freeman BE Mansfield JM. Modulation of innate immunity by African Trypanosomes. Parasitology. (2010) 137:2051–63. 10.1017/S0031182010001460 21087532

[7] Rock RB Gekker G Hu S Sheng WS Cheeran M Lokensgard JR. Role of microglia in central nervous system infections [Internet]. Clin Microbiol Rev. (2004) 17:942–64. 10.1128/CMR.17.4.942-964.2004 15489356 PMC523558

[8] Lemos KR Marques LC Aquino LPCT Alessi AC Zacarias RZ. Astrocytic and microglial response and histopathological changes in the brain of horses with experimental chronic *Trypanosoma evansi* infection. Rev Inst Med Trop São Paulo. (2008) 50:243–9. 10.1590/S0036-46652008000400011 18813766

[9] Chianella S Semprevivo M Peng ZC Zaccheo D Bentivoglio M Grassi-Zucconi G. Microglia activation in a model of sleep disorder: an immunohistochemical study in the rat brain during *Trypanosoma brucei* infection. Brain Res. (1999) 832:54–62. 10.1016/S0006-8993(99)01449-3 10375652

[10] Bentivoglio M Kristensson K. Tryps and trips: cell trafficking across the 100-year-old blood-brain barrier. Trends Neurosci. (2014) 37: 325–33. 10.1016/j.tins.2014.03.007 24780507 PMC4045197

[11] Paine A Eiz-Vesper B Blasczyk R Immenschuh S. Signaling to heme oxygenase-1 and its anti-inflammatory therapeutic potential. Biochem Pharmacol. (2010) 80:1895–903. 10.1016/j.bcp.2010.07.014 20643109

[12] Lee T-S Chau L-Y. Heme oxygenase-1 mediates the anti-inflammatory effect of interleukin-10 in mice. Nat Med. (2002) 8:240–6. 10.1038/nm0302-240 11875494

[13] Jung S-S Moon J-S Xu J-F Ifedigbo E Ryter SW Choi AMK. Carbon monoxide negatively regulates NLRP3 inflammasome activation in macrophages. Am J Physiol. (2015) 308:L1058–67. 10.1152/ajplung.00400.2014 25770182 PMC4437010

[14] Morse D Pischke SE Zhou Z Davis RJ Flavell RA Loop T. Suppression of inflammatory cytokine production by carbon monoxide involves the JNK pathway and AP-1. J Biol Chem. (2003) 278:36993–8. 10.1074/jbc.M302942200 12857751

[15] Sedlak TW Saleh M Higginson DS Paul BD Juluri KR Snyder SH. Bilirubin and glutathione have complementary antioxidant and cytoprotective roles. Proc Natl Acad Sci USA. (2009) 106:5171–6. 10.1073/pnas.0813132106 19286972 PMC2664041

[16] Epiphanio S Mikolajczak SA Gonçalves LA Pamplona A Portugal S Albuquerque S. Heme oxygenase-1 is an anti-inflammatory host factor that promotes murine plasmodium liver infection. Cell Host Microbe. (2008) 3:331–8. 10.1016/j.chom.2008.04.003 18474360

[17] Carasi P Rodríguez E da Costa V Frigerio S Brossard N Noya V. Heme-Oxygenase-1 expression contributes to the immunoregulation induced by *Fasciola hepatica* and promotes infection. Front Immunol. (2017) 8:883. 10.3389/fimmu.2017.00883 28798750 PMC5526848

[18] Luz NF Andrade BB Feijó DF Araújo-Santos T Carvalho GQ Andrade D. Infection leishmania chagasi of heme oxygenase-1 promotes the persistence heme oxygenase-1 promotes the persistence of leishmania chagasi infection. J Immunol. (2018) 188: 4460–7. 10.4049/jimmunol.1103072 22461696 PMC3331931

[19] Gutierrez F Pavanelli W Medina T Silva G Mariano F Guedes P Heme Oxygenase activity protects the host against excessive cardiac inflammation during experimental *Trypanosoma cruzi* infection. Microbes Infect. (2013) 16:28–39. 10.1016/j.micinf.2013.10.007 24140555

[20] Namangala B De Baetselier P Brijs L Stijlemans B Noël W Pays E. Attenuation of *Trypanosoma brucei* is associated with reduced immunosuppression and concomitant production of Th2 lymphokines. J Infect Dis. (2000) 181:1110–20. 10.1086/315322 10720538

[21] Stibbs HH Seed JR. Chromatographic evidence for the synthesis of possible sleep-mediators in*Trypanosoma brucei* gambiense. Experientia. (1973) 29:1563–5. 10.1007/BF01943919 4543926

[22] Seed JR Hall JE Sechelski J. Phenylalanine metabolism in *Microtus montanus* chronically infected with *Trypanosoma brucei* gambiense. Comp Biochem Physiol. (1982) 71: 209–15. 10.1016/0305-0491(82)90242-5 7037281

[23] Newport GR Page CR Ashman PU Stibbs HH Seed JR. Alteration of free serum amino acids in voles infected with *Trypanosoma brucei* gambiense. J Parasitol. (1977) 63:15–24. 10.2307/3280098 321737

[24] Hall JE Seed JR Sechelski JB. Multiple alpha-keto aciduria in *Microtus montanus* chronically infected with *Trypanosoma brucei* gambiense. Comp Biochem Physiol. (1985) 82: 73–8. 10.1016/0305-0491(85)90130-0 3902349

[25] El Sawalhy A Seed JR Hall JE El Attar H. Increased excretion of aromatic amino acid catabolites in animals infected with *Trypanosoma brucei evansi*. J Parasitol. (1998) 84:469–73. 10.2307/3284707 9645841

[26] Marciano D Llorente C Maugeri DA de la Fuente C Opperdoes F Cazzulo JJ. Biochemical characterization of stage-specific isoforms of aspartate aminotransferases from *Trypanosoma cruzi* and *Trypanosoma brucei*. Mol Biochem Parasitol. (2008) 161:12–20. 10.1016/j.molbiopara.2008.05.005 18602174

[27] Berger BJ Dai WW Wang H Stark RE Cerami A. Aromatic amino acid transamination and methionine recycling in trypanosomatids. Proc Natl Acad Sci USA. (1996) 93:4126–30. 10.1073/pnas.93.9.4126 8633027 PMC39498

[28] Cazzulo Franke MC Vernal J Cazzulo JJ Nowicki C. The NAD-linked aromatic α-hydroxy acid dehydrogenase from *Trypanosoma cruzi*. A new member of the cytosolic malate dehydrogenases group without malate dehydrogenase activity. Eur J Biochem. (1999) 266:903–10. 10.1046/j.1432-1327.1999.00926.x 10583384

[29] Nowicki C Cazzulo JJ. Aromatic amino acid catabolism in trypanosomatids. Comp Biochem Physiol. (2008) 151:381–90. 10.1016/j.cbpa.2007.03.010 17433885

[30] McGettrick AF Corcoran SE Barry PJG McFarland J Crès C Curtis AM. *Trypanosoma brucei* metabolite indolepyruvate decreases HIF-1α and glycolysis in macrophages as a mechanism of innate immune evasion. Proc Natl Acad Sci USA. (2016) 113:E7778–87. 10.1073/pnas.1608221113 27856732 PMC5137691

[31] Murphy S Simmons ML Agullo L Garcia A Feinstein DL Galea E. Synthesis of nitric oxide in CNS glial cells. Trends Neurosci. (1993) 16:323–8. 10.1016/0166-2236(93)90109-Y 7691008

[32] MacMicking J Xie Q Nathan C. Nitric oxide and macrophage function. Annu Rev Immunol. (1997) 15:323–50. 10.1146/annurev.immunol.15.1.323 9143691

[33] Mucha O Podkalicka P Czarnek M Biela A Mieczkowski M Kachamakova-Trojanowska N. Pharmacological versus genetic inhibition of heme oxygenase-1 - the comparison of metalloporphyrins, shRNA and CRISPR/Cas9 system. Acta Biochim Pol. (2018) 65:277–86. 10.18388/abp.2017_2542 29694447

[34] Ma Q. Role of Nrf2 in oxidative stress and toxicity. Annu Rev Pharmacol Toxicol. (2013) 53:401–26. 10.1146/annurev-pharmtox-011112-140320 23294312 PMC4680839

[35] Stijlemans B Beschin A Magez S Van Ginderachter JA De Baetselier P. Iron homeostasis and *Trypanosoma brucei* associated immunopathogenicity development: a battle/quest for iron. Biomed Res Int. (2015) 2015:819389. 10.1155/2015/819389 26090446 PMC4450282

[36] Kořený L Oborník M Lukeš J. Make It, Take it, or leave it: heme metabolism of parasites. PLoS Pathog. (2013) 9:e1003088. 10.1371/journal.ppat.1003088 23349629 PMC3547853

[37] Garzón E Holzmuller P Bras-Gonçalves R Vincendeau P Cuny G Lemesre JL. The *Trypanosoma brucei* gambiense secretome impairs lipopolysaccharide-induced maturation, cytokine production, and allostimulatory capacity of dendritic cells. Infect Immun. (2013) 81:3300–8. 10.1128/IAI.00125-13 23798533 PMC3754197

[38] Quan N He L Lai W Intraventricular infusion of antagonists of IL-1 and TNFα attenuates neurodegeneration induced by the infection of *Trypanosoma brucei*. J Neuroimmunol. (2003) 138:92–8. 10.1016/S0165-5728(03)00122-X 12742658

[39] Chora ÂA Fontoura P Cunha A Pais TF Cardoso S Ho PP. Heme oxygenase−1 and carbon monoxide suppress autoimmune neuroinflammation. J Clin Invest. (2007) 117:438–47. 10.1172/JCI28844 17256058 PMC1770945

[40] Chen-Roetling J Kamalapathy P Cao Y Song W Schipper HM Regan RF. Astrocyte heme oxygenase-1 reduces mortality and improves outcome after collagenase-induced intracerebral hemorrhage. Neurobiol Dis. (2017) 102:140–6. 10.1016/j.nbd.2017.03.008 28323022 PMC5604234

[41] Soares MP Ribeiro AM. Nrf2 as a master regulator of tissue damage control and disease tolerance to infection. Biochem Soc Trans. (2015) 43:663–8. 10.1042/BST20150054 26551709 PMC4613525

[42] Paiva CN Feijó DF Dutra FF Carneiro VC Freitas GB Alves LS. Oxidative stress fuels *Trypanosoma cruzi* infection in mice. J Clin Invest. (2012) 122:2531–42. 10.1172/JCI58525 22728935 PMC3386808

[43] Aoki R Aoki-Yoshida A Suzuki C Takayama Y. Protective effect of indole-3-pyruvate against ultraviolet b-induced damage to cultured HaCaT keratinocytes and the skin of hairless mice. PLoS ONE. (2014) 9:e96804. 10.1371/journal.pone.0096804 24810606 PMC4014565

[44] Fernando PMDJ Piao MJ Kang KA Ryu YS Hewage SRKM Chae SW. Rosmarinic acid attenuates cell damage against UVB radiation-induced oxidative stress via enhancing antioxidant effects in human HaCaT cells. Biomol Ther. (2016) 24:75–84. 10.4062/biomolther.2015.069 26759705 PMC4703356

[45] Schäfer M Dütsch S auf dem Keller U Navid F Schwarz A Johnson DA. Nrf2 establishes a glutathione-mediated gradient of UVB cytoprotection in the epidermis. Genes Dev. (2010) 24:1045–58. 10.1101/gad.568810 20478997 PMC2867209

[46] Saw CL Huang MT Liu Y Khor TO Conney AH Kong AN. Impact of Nrf2 on UVB-induced skin inflammation/photoprotection and photoprotective effect of sulforaphane. Mol Carcinog. (2011) 50:479–86. 10.1002/mc.20725 21557329

