# Supplementary material for: Trypanosoma brucei Secreted Aromatic Ketoacids Activate the Nrf2/HO-1 Pathway and Suppress Pro-inflammatory Responses in Primary Murine Glia and Macrophages
Source: Front Immunol. 2019 Sep 11;10:2137. doi: 10.3389/fimmu.2019.02137 (PMC6749089; doi:10.3389/fimmu.2019.02137)
Supplement: Supplementary file 1 [file Data_Sheet_1.pdf]

Supplementary Material

Supplementary Figures

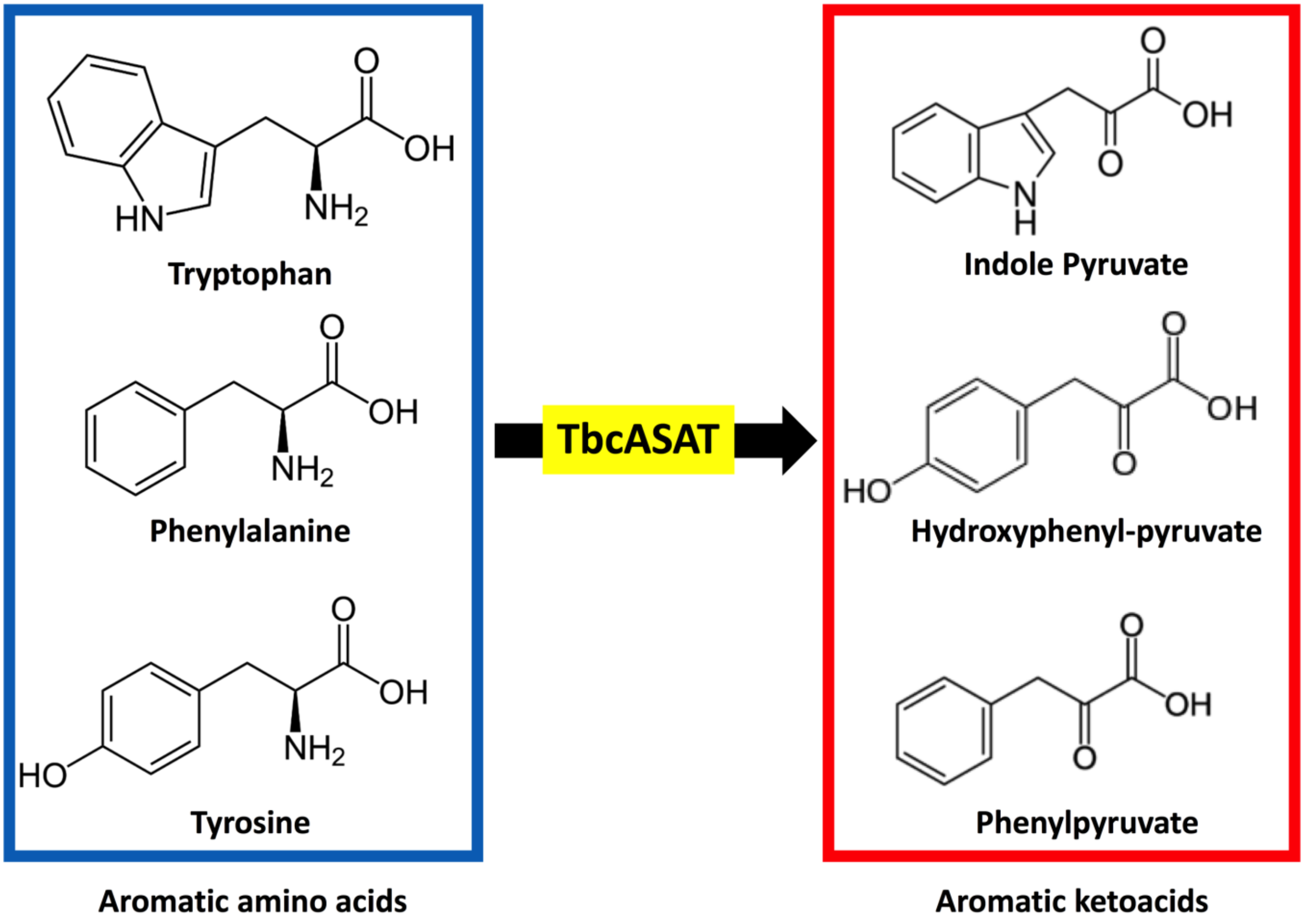

Supplementary Figure 1. Structures of aromatic ketoacids produced by *T. brucei* cytoplasmic aspartate aminotransferase. Schematic illustrating the transamination of aromatic amino acids (tryptophan, phenylalanine, tyrosine) to aromatic ketoacids (indole pyruvate, hydroxyphenylpyruvate and phenylpyruvate) by *T. brucei* cytoplasmic aspartate aminotransferase (TbcASAT).

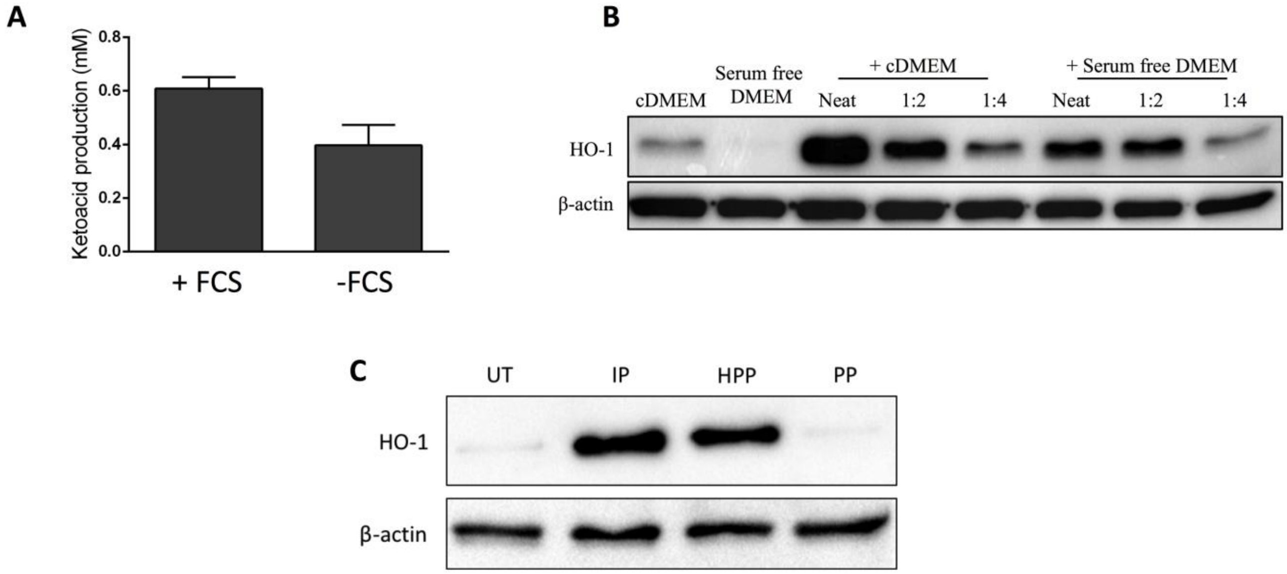

Supplementary Figure 2. Removal of 10% FCS supplement leads to lower secreted ketoacid levels, while maintaining HO-1 induction. (A) Ketoacid production was optimal in the presence of FCS (+FCS) and decreased by 35% when FBS was absent from the culture media (-FCS). Graph of total ketoacids (mM) detected in the media after 5 hours. Wild type cells were cultured at high density (5 x10^7 cells/ml) in media with or without 10% FCS, supplemented with 1 mM each of tryptophan, phenylalanine, and tyrosine. AHADH assay was performed to measure ketoacid production. **(B)** Neat or diluted (1:2 or 1:4) supernatant from T. brucei cultures was added to primary murine mixed glia. Expression of HO-1 was measured after 24 hours by Western blot. **(C)** Primary murine BMDM were cultured in complete media, cells were then washed, and media was replaced with serum free media to which purified ketoacids (IP, HPP and PP) were added. After 24 hours expression of HO-1 was measured by Western blot. Full length blots are presented in Supplementary Figure 8 A & B.

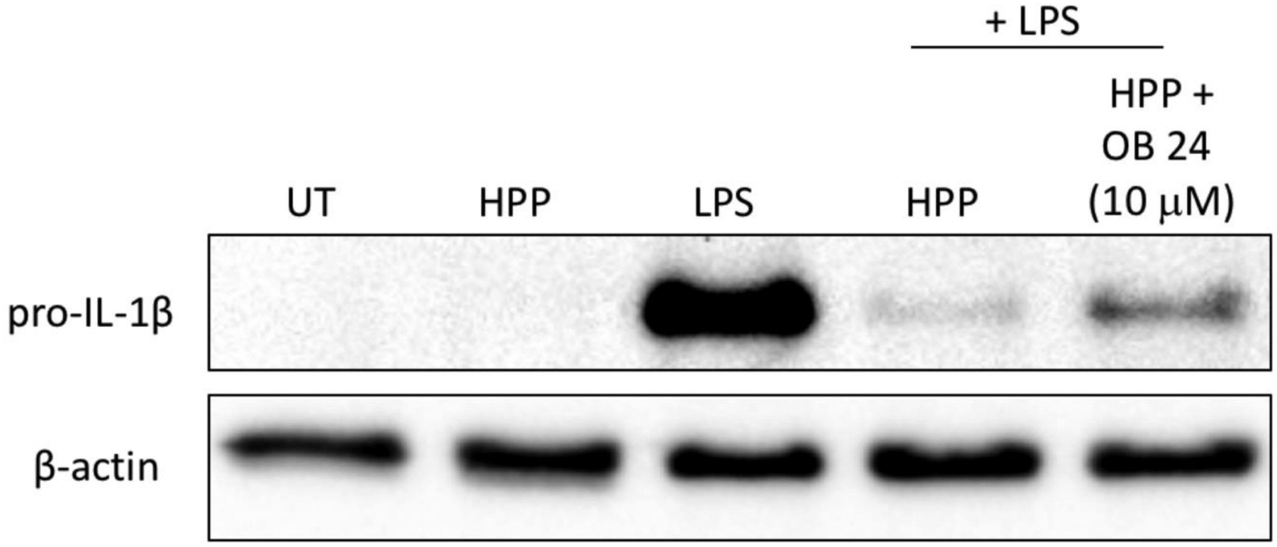

Supplementary Figure 3. Inhibition of HO-1 partially restores LPS-induced pro-IL-1β expression in HPP-treated BMDM. Primary murine BMDM were pre-treated with the specific HO-1 inhibitor, OB 24 hydrochloride (10 μM), for 60 minutes, followed by treatment with HPP (1 mM) for 30 minutes, prior to stimulation with LPS (100 ng/ml) for 24 hours. Expression of pro-IL-1β was measured by Western blot. Full length blots are shown in Supplementary Figure 8 C.

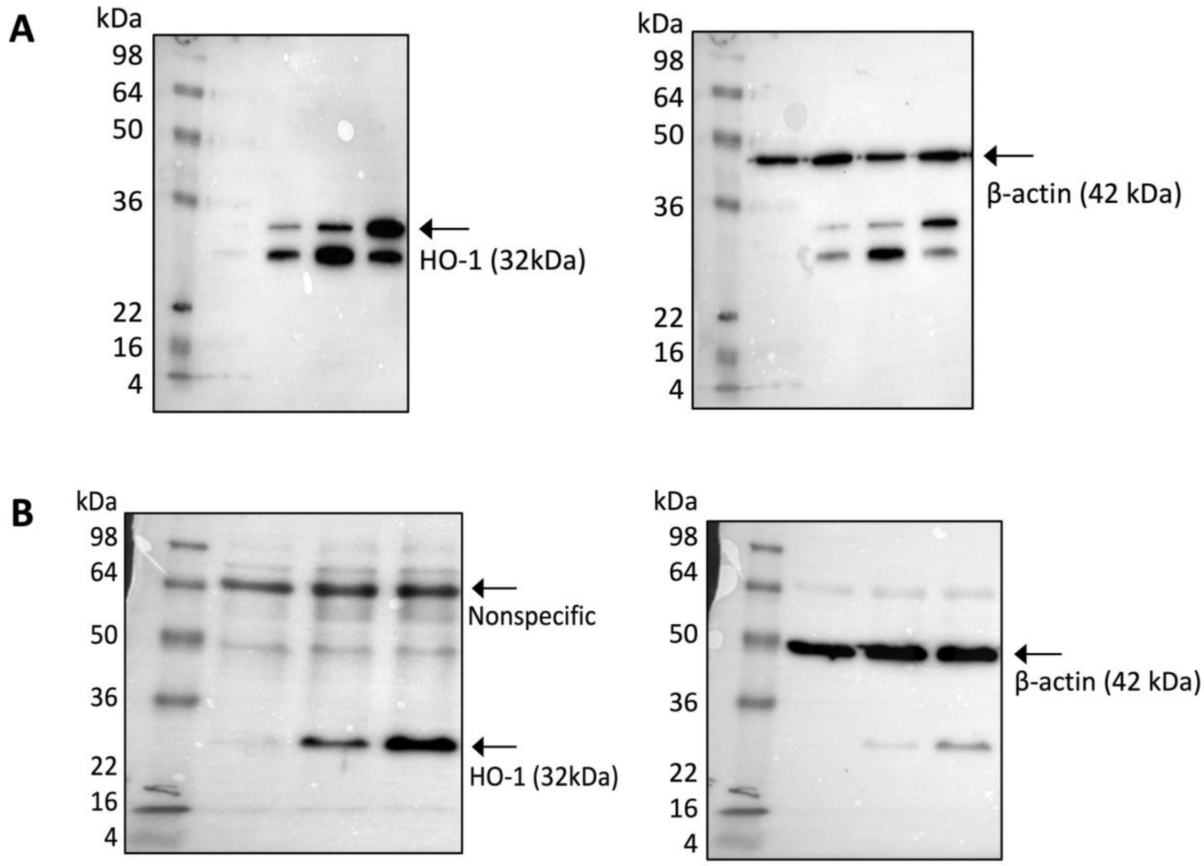

Supplemental Figure 4. Full length blots for Figure 1A and 1B. HO-1 expression in murine mixed glia was measured by western blot. The membranes were then re-probed for β-actin as a loading control. Blots were developed using enhanced chemiluminescent substrate with a BioRad ChemiDoc MP system. Full scan images of the cropped exposure images used in Figure 1A **(A)** and Figure 1B **(B)** are merged with the protein ladder to show molecular weights of the proteins. Nonspecific binding of antibodies is highlighted in the image where relevant. HO-1 often appears as a doublet, this faster migrating form of HO-1 is described by Lin *et al*; 'Heme Oxygenase-1 Protein Localizes to the Nucleus and Activates Transcription Factors Important in Oxidative Stress', *J.Biol.Chem*. (2007) 282, 20621-20633.

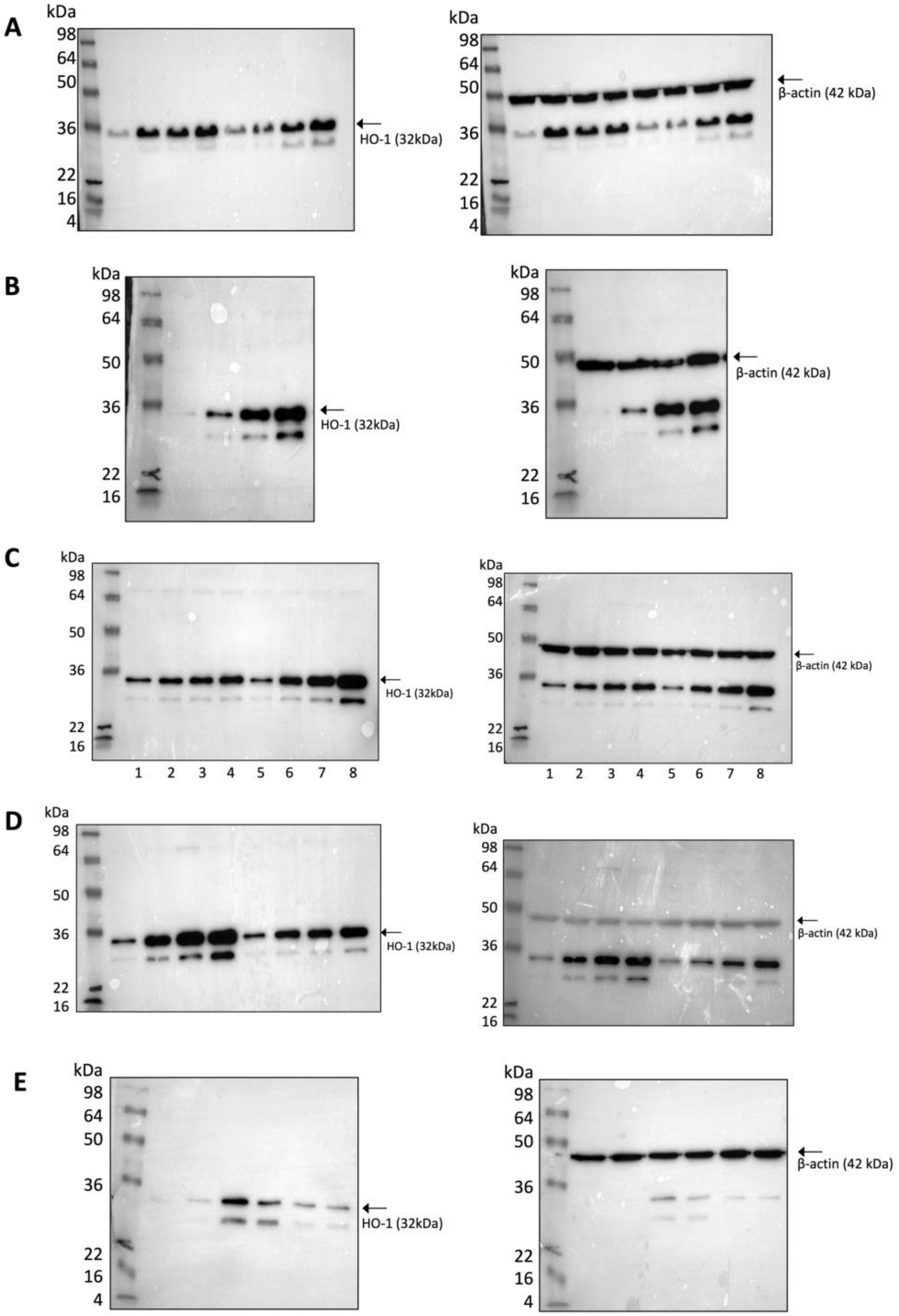

Supplemental Figure 5. Full length blots for Figure 2B, 2D and 2H. HO-1 expression in murine mixed glia and BMDM were measured by western blot. The membranes were then re-probed for β-actin as a loading control. Blots were developed using enhanced chemiluminescent substrate with a BioRad ChemiDoc MP system. Full scan images of the cropped exposure images used in Figure 2B **(A & B),** Figure 2D **(C & D)** and Figure 2H **(E)** are merged with the protein ladder to show molecular weights of the proteins. Lanes 1 - 4 in Figure C contain unrelated experimental samples.

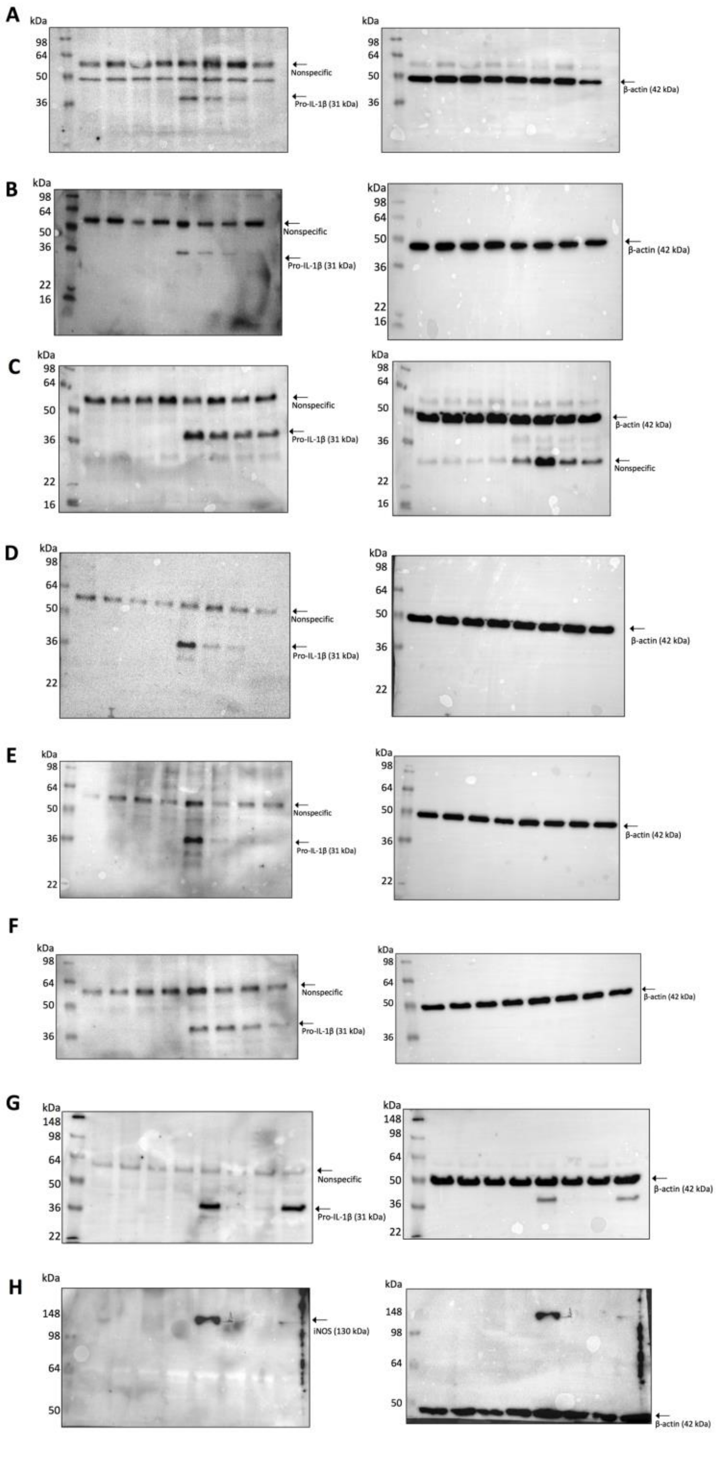

Supplemental Figure 6. Full length blots for Figure 3B, 3B, 3C and 3D. Pro-IL-1β expression in murine mixed glia, microglia and BMDM, and iNOS expression in BMDM were measured by western blot. The membranes were then re-probed for β-actin as a loading control. Blots were developed using enhanced chemiluminescent substrate with a BioRad ChemiDoc MP system. Full scan images of the cropped exposure images used in Figure 3A **(A & B & C),** Figure 3B **(D & E & F),** Figure 3C **(G)** and Figure 3D **(F)** are merged with the protein ladder to show molecular weights of the proteins. Nonspecific binding of antibodies is highlighted in the image where relevant.

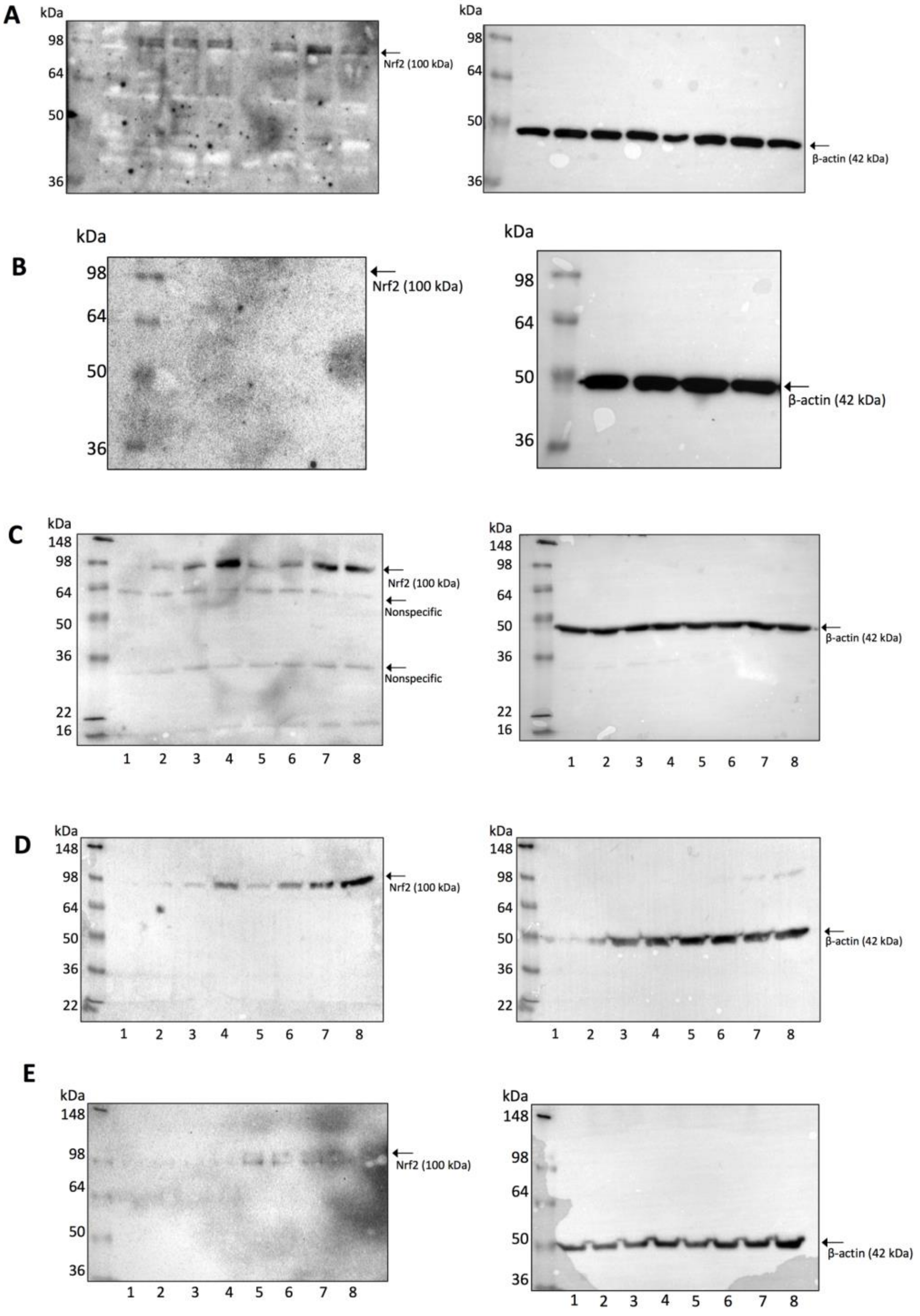

Supplemental Figure 7. Full length blots for Figure 5A and 5B. Nrf2 expression in murine mixed glia and BMDM were measured by western blot. The membranes were then re-probed for β-actin as a loading control. Blots were developed using enhanced chemiluminescent substrate with a BioRad ChemiDoc MP system. Full scan images of the cropped exposure images used in Figure 5A **(A & B)** and Figure 5B **(C & D & E)** are merged with the protein ladder to show molecular weights of the proteins. Lanes 1 - 4 in Figures C, D and E contain unrelated experimental samples. Nonspecific binding of antibodies is highlighted in the image where relevant.

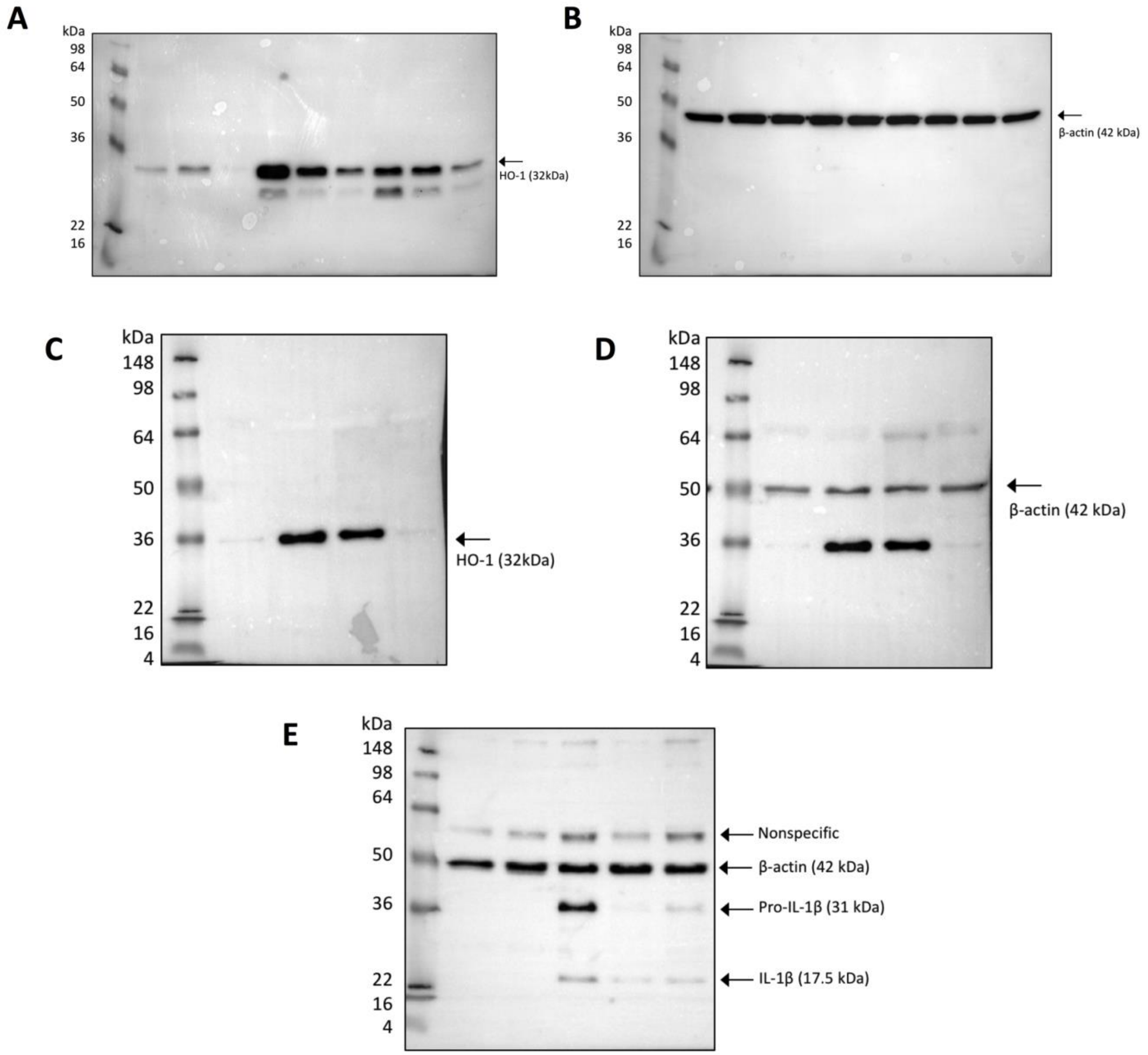

Supplementary Figure 8. Full length blots for Supplementary Figures 2 & 3. HO-1 and pro-IL-1β expression in murine mixed glia and BMDM were measured by western blot. The membranes were then re-probed for β-actin as a loading control. Blots were developed using enhanced chemiluminescent substrate with a BioRad ChemiDoc MP system. Full scan images of the cropped exposure images used in Supplementary Figure 2B **(A & B),** Supplementary Figure 2C **(C & D)** and Supplementary Figure 3 **(E)** are merged with the protein ladder to show molecular weights of the proteins. Lane 1 in Figure A & B contain unrelated experimental samples.
